# Heterogeneous structural changes correlated to local atomic order in thermal rejuvenation process of Cu-Zr metallic glass

**DOI:** 10.1080/14686996.2019.1624140

**Published:** 2019-06-19

**Authors:** Masato Wakeda, Junji Saida

**Affiliations:** a Research Center for Structural Materials, National Institute for Materials Science, Ibaraki, Japan; b Frontier Research Institute for Interdisciplinary Sciences, Tohoku University, Miyagi, Japan

**Keywords:** Metallic glass, rejuvenation, structural heterogeneity, molecular dynamics, geometrical structure, 10 Engineering and Structural materials, 106 Metallic materials, 107 Glass and ceramic materials, 400 Modeling / Simulations

## Abstract

In this study, we investigated the atomistic mechanism of structural excitation in a thermal process (thermal rejuvenation) of metallic glass. In a molecular dynamics framework, Cu-Zr metallic glass was rejuvenated by a thermal process composed of an isothermal heat treatment at a temperature above the glass transition temperature $T_{g}$, followed by fast cooling. Atomistic analyses of the local rearrangement, potential energy, and geometrical structure revealed structural changes correlating to the local atomic order in the rejuvenation process. In the early stage of the heat treatment for thermal rejuvenation, the structural excitation exhibited spatial heterogeneity at the nanometer scale. More-excited regions (i.e., regions with large atomic non-affine and affine transformations) exhibited low-ordered structures and vice versa, implying that the local structural excitation is significantly correlated with the local atomic order. The structural excitation transitioned from partial to whole as the isothermal process proceeded above $T_{g}$. Although rejuvenation decreased the ordered structure, the calculation results suggested the formation of newly ordered local structures and newly disordered local structures correlated to local structural excitations and atomic dynamics in the thermal process. These results indicate that the heterogeneous structure evolution of the rejuvenation process induces a redistribution of the local atomic order in the microstructure of metallic glasses.

## Graphical abstract

**Figure:**
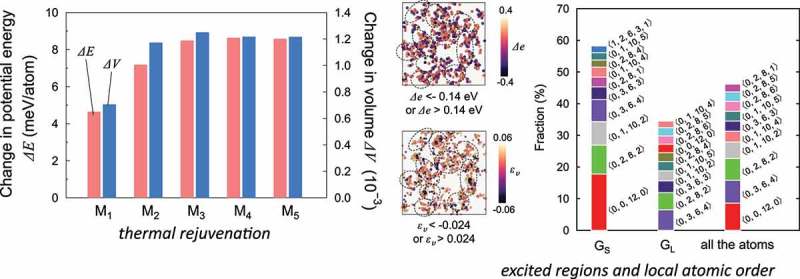

## 1. Introduction

Metallic glass exhibits fascinating properties, such as large elastic elongation, high fracture toughness, high fatigue strength, high hardness, and high corrosion resistance [1,2]. Rejuvenation has attracted recent attention in the metallic glass field [3,4]. It is the structural excitation of metallic glass, which induces a lower density and higher potential energy. Therefore, it is an opposite phenomenon to aging from the aspects of density and potential energy. Rejuvenation is available to control the relaxation state of metallic glasses [3,5]. Recently, various rejuvenation processes of metallic glass, such as shot-peening [6], high-pressure torsion [7], and thermal cycles at low temperature [8], have been proposed. Structural excitation by a thermal process at a temperature above the glass transition temperature, $T_{g}$, is a rejuvenation technique [5,9,10]. In our previous studies, we presented feasibility conditions for thermal rejuvenation using computational and experimental approaches [5,10,11]. We demonstrated that rejuvenation could be realized by increasing the temperature above 1.1$T_{g}$, followed by subsequent quenching with faster cooling compared with a previous quenching process; the former process was required for realizing a high-energy state (or erasing thermal history), while the latter was required for suppressing relaxation during the quenching process. Meanwhile, the structural and atomistic origins of thermal rejuvenation are still unclear; the question remains on where and how thermal structural excitation occurs in metallic glasses. Nanoscale heterogeneity in the microstructure of metallic glasses has been emphasized in recent experimental and computational studies [12–17], because heterogeneity is crucial in deformation, crystallization, and relaxation behaviors. Some studies suggested that characteristic local regions so-called weakly bonding regions (or liquid like regions, loosely packed regions) and strongly bonding regions (or closely packed regions) in metallic glasses exist [15,18]. The local atomic order, such as geometrical local clusters, also has heterogeneity at the nanometer scale [14,19]. Atomistic insights into the rejuvenation process should provide useful information to control the structures and properties of metallic glasses, and also enhance a deeper understanding of structural features in metallic glasses. In this study, we provide atomistic insights into the structural excitation of metallic glasses in the isothermal process above $T_{g}$, particularly focusing on the correlation between the structural heterogeneity and excitation regions. Using molecular dynamics (MD) simulations, we constructed a relaxed glass model of a Cu-Zr binary alloy, and subsequently conducted a thermal process for rejuvenation. Based on the analyses of atomic non-affine and affine transformations, squared displacement, potential energy, and geometrical structures, we revealed the atomistic mechanism of structural excitation in the thermal rejuvenation process.

## 2. Methods

**Figure 1.**
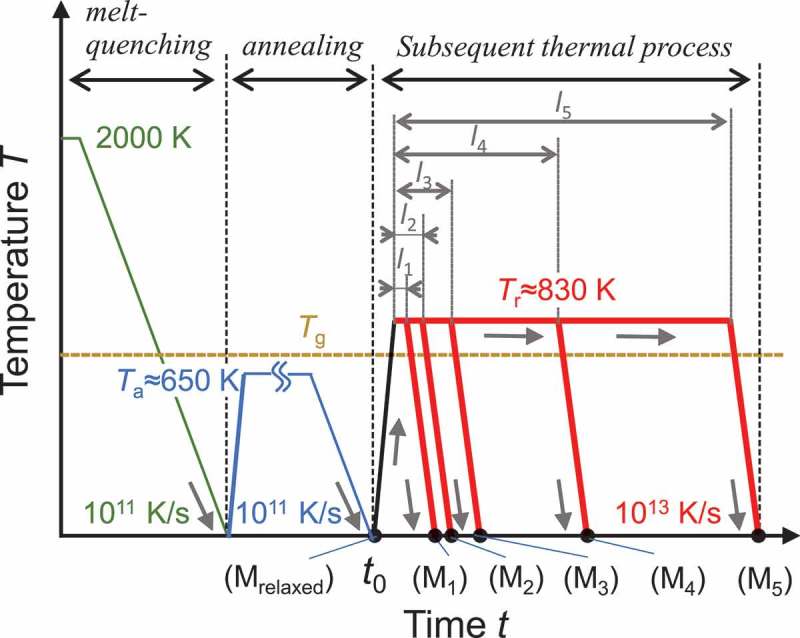
Schematics of melt-quenching, annealing, and subsequent thermal processes. M${\;}_{relaxed}$ is the model after annealing. M${\;}_{1}$, M${\;}_{2}$, M${\;}_{3}$, M${\;}_{4}$, and M${\;}_{5}$ are the models after the subsequent thermal process, in which the isothermal holding times are 0.5, 1.0, 2.0, 5.0, and 10.0 ns, respectively.

In this study, MD techniques based on the semi-empirical potential developed for Cu-Zr alloys [20] were used. We constructed a relaxed glass model and rejuvenated glass models based on the heat treatment proposed in our previous study [5]. First, a Cu${\;}_{60}$Zr${\;}_{40}$ glass model was prepared through melt-quenching from a high-temperature liquid (2000 K) to glass solid (1 K) at a constant cooling rate of 10${\;}^{11}$ K/s. The number of atoms, $N_{a}$, was 50,000. From a change in slope of the volume-temperature curve in the melt-quenching process, the glass transition temperature, $T_{g}$ (∼730 K) was determined. The quenched model was subsequently annealed at 650 K ($<T_{g}$) for 10.0 ns and cooled again at 10${\;}^{11}$ K/s. A cooling rate of 10${\;}^{11}$ K/s is relatively low for the MD simulations. The constructed model, after being annealed, is denoted by M${\;}_{relaxed}$ (hereinafter ‘relaxed model’). For the relaxed model, we conducted thermal rejuvenation according to the following process. The starting time of the subsequent thermal process is denoted by $t_{0}$. First, the relaxed model was reheated to 830 K at a heating rate of 10${\;}^{13}$ K/s, and underwent an isothermal heat treatment at 830 K, which was higher than $T_{g}$. After the isothermal process the model was quenched to 1 K at 10${\;}^{13}$ K/s, which was much higher than the previous cooling rate, 10${\;}^{11}$ K/s. To obtain different degrees of rejuvenation, we employed five different holding times of isothermal processes: $l_{1}=0.5$, $l_{2}=1.0$, $l_{3}=2.0$, $l_{4}=5.0$, and $l_{5}=10.0$ ns. The models after the subsequent thermal processes with $l_{1}$–$l_{5}$ are denoted by M${\;}_{1}$–M${\;}_{5}$, respectively. Figure 1 shows the schematics of the initial melt-quenching, annealing, and subsequent thermal processes. All thermal processes were conducted under an isothermal-isobaric ensemble with a zero-pressure condition, and periodic boundary conditions were applied in all orthogonal directions. The relaxed and rejuvenated models have $T_{g}$ different from that of the as-quenched model, owing to their different relaxation-state degrees.

## 3. Results

### 3.1. Changes in potential energy and volume

**Figure 2.**
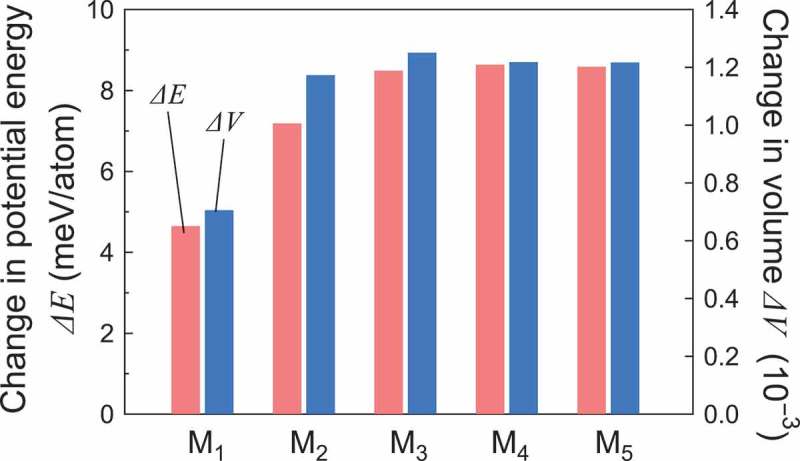
Changes in potential energy, ΔE, and volume, ΔV, by the subsequent thermal process. M${\;}_{1}$–M${\;}_{5}$ correspond to models M${\;}_{1}$–M${\;}_{5}$ in Figure 1, respectively.

Changes in the potential energy, ΔE, and volume, ΔV, owing to the subsequent thermal process are summarized in Figure 2. ΔE and ΔV are defined by $\left(E(M_{i})-E(M_{relaxed})\right)/N_{a}$ and $\left(V(M_{i})-V(M_{relaxed})\right)/V(M_{relaxed})$ (i=1–5), respectively. For each $l_{i}$, we conducted three independent simulations with different initial atomic velocities, and confirmed that the initial velocity had a negligibly small effect on the final potential energy and volume. Figure 2 shows the averaged result of the three independent simulations. Because rejuvenation increases both potential energy and volume, as mentioned above, positive ΔE and ΔV represent the degree of rejuvenation, while their negative values represent the degree of aging. In all $l_{i}\left(i=1\right.$–$\left.5\right)$, we observed rejuvenation (i.e., positive changes in potential energy and volume), because the subsequent thermal process satisfied the conditions necessary for thermal rejuvenation [5,10,11]. Because the temperature of the isothermal process was slightly greater than $T_{g}$, thermal excitation occurred gradually over the MD timescale. Therefore, M${\;}_{1}$–M${\;}_{3}$ demonstrated that the degree of rejuvenation depended on the holding time of the isothermal process in this thermal condition; a longer isothermal process induced a higher degree of rejuvenation, and vice versa. Meanwhile, M${\;}_{3}$–M${\;}_{5}$ demonstrated that the degree of rejuvenation saturated if the isothermal process was sufficiently long.

### 3.2. Local structural rearrangements in early stage of the thermal process

**Figure 3.**
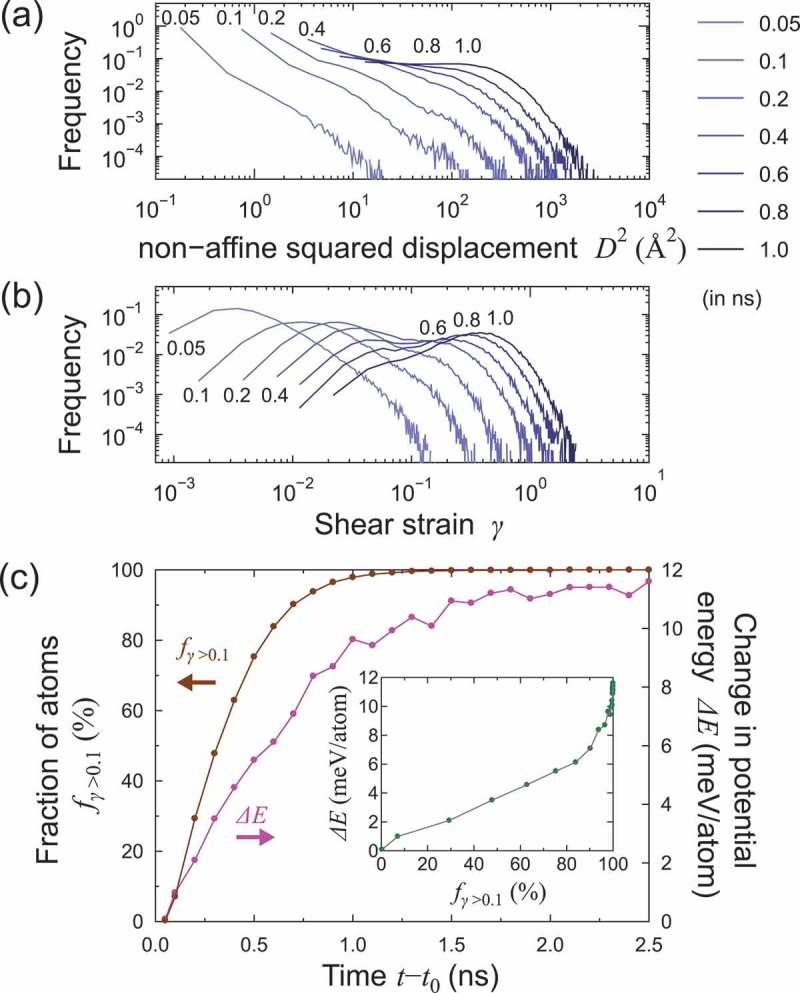
Histogram of the atomic (a) non-affine squared displacement $D^{2}$ and (b) shear strain γ in the early stage of the subsequent thermal process $t-t_{0}\leq 1.0$ ns (0.05, 0.1, 0.2, 0.4, 0.6, 0.8, and 1.0 ns). The potential energy evolution, ΔE, and the change in the fraction of atoms with a atomic shear strain greater than 0.1, $f_{\gamma >0.1}$, are shown in panel (c). The inset plot shows the relationship between ΔE and $f_{\gamma >0.1}$.

First, we provide atomistic insights into the early stage of the thermal process ($t-t_{0}\leq 2.5$ ns). Based on atomic strain analyses [21,22], the non-affine squared displacement $D^{2}$, von Mises local shear invariant (shear strain, hereafter) γ, and volumetric strain $\varepsilon_{\upsilon }$ of each of the atoms were calculated [23]. The reference atomic configuration for the calculations is the initial structure of the subsequent thermal process (i.e., M${\;}_{relaxed}$). To remove the effects of atomic thermal fluctuations on the calculation, we used atomic structures quenched from snapshots in the subsequent thermal process. The quenching was conducted by the conjugate gradient method at 0 K; during the quenching process, the model size was controlled to maintain a zero-pressure condition. All the results shown hereafter were based on the quenched atomic configurations except for shear simulations. Histograms of the non-affine squared displacement and shear strain for $0.05\leq t-t_{0}\leq 1.0$ ns are shown in Figure 3(a,b), respectively. Non-affine squared displacement has been used for the discussion of local rearrangement in deformation [21] and a precursor of crystallization [24] in metallic glasses. The non-affine squared displacement and shear strain increased as the thermal process proceeded, implying that both the local non-affine and affine transformations were activated gradually in the heating and isothermal processes.

The histograms of the atomic shear strain exhibited two peaks, $0.4\leq t-t_{0}\leq 0.8$ ns, and the mid-point between the two peaks corresponded to γ≈0.1. The two peaks imply that a critical atomic shear strain or a characteristic energy barrier existed for the shear transformation. Figure 3(c) shows the time evolution of the fraction of atoms with an atomic shear strain greater than 0.1, denoted by $f_{\gamma >0.1}$. The change in potential energy of the quenched model is also shown. $f_{\gamma >0.1}$ increased rapidly during the isothermal process, reaching 100% at $t-t_{0}∼1.5$ ns. Therefore, in terms of the atomic shear strain and potential energy, structural excitation (or local melting) proceeded in the early stage of the isothermal process ($t-t_{0}\leq 2.0$ ns).

**Figure 4.**
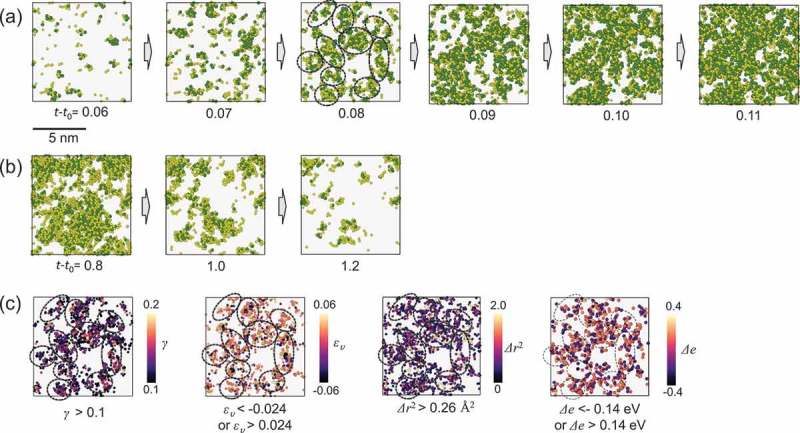
Spatial distributions of atoms with (a) large $D^{2}$ for $0.06\leq t-t_{0}\leq 0.11$ ns and (b) small $D^{2}$ for $0.8\leq t-t_{0}\leq 1.2$ ns. In panels (a) and (b), $D^{2}=7$${\text{\textbackslash{}Aring}}^{2}$ is a threshold value. The time, $t-t_{0}$, is given below each figure. The lighter and darker atoms are Cu and Zr, respectively. (c) Atoms with significant atomic shear strain γ, volumetric strain $\varepsilon_{\upsilon }$, atomic squared displacement $\Delta r^{2}$, and change in potential energy Δe at $t-t_{0}=0.08$ ns. In panel (c), the threshold values for the visualizations are denoted below each plot, and the color bars are shown on the right. The dotted circles in panels (a) and (c) for the models at $t-t_{0}=0.08$ ns show similarities in the distributions of atoms with large $D^{2}$, shear strain γ, volumetric strain $\varepsilon_{\upsilon }$, atomic squared displacement $\Delta r^{2}$, and change in potential energy Δe.

Figure 4(a) shows the spatial distribution of the atoms with a large non-affine squared displacement, $D^{2}$, for $0.06\leq t-t_{0}\leq 0.11$ ns. Here we used $D^{2}$ to evaluate structural excitations in metallic glasses; Figure 4(a) shows more-excited regions in the very early stage of the subsequent thermal process. In addition, Figure 4(b) shows atoms with small $D^{2}$ (i.e., atoms in the less-excited regions) for $0.8\leq t-t_{0}\leq 1.2$ ns. At $t-t_{0}=0.06$ ns in Figure 4(a), atoms with large $D^{2}$ were not randomly distributed but formed clusters, and the cluster sizes increased rapidly during the isothermal process. The less-excited atoms in Figure 4(b) also formed clusters, and the cluster size decreased as the isothermal process proceeded. This result reveals that thermal structural excitation results in spatial heterogeneity at the nanometer scale. Because rejuvenation accompanies the erasing of the relaxation state, heterogeneity in thermal rejuvenation implies heterogeneity in thermal stability against the erasing process. Figure 4(c) shows atoms with a large atomic shear strain γ, volumetric strain $\varepsilon_{\upsilon }$, squared displacement $\Delta r^{2}$, and a change in potential energy Δe at $t-t_{0}=0.08$ ns. Regarding the calculation of γ, $\varepsilon_{\upsilon }$, $\Delta r^{2}$, and Δe, a reference state is the initial structure of the subsequent thermal process, M${\;}_{relaxed}$. It is clear that the distribution of atoms with large $D^{2}$ corresponds to that of atoms with large γ, $\varepsilon_{\upsilon }$, $\Delta r^{2}$, and Δe. Therefore, the thermally excited regions exhibited both large components of non-affine and affine rearrangements and induced local potential energy and volume changes. It is noteworthy that the potential energy change and volumetric strain of individual atoms were both positive and negative, although the macroscopic (i.e., average) potential energy and volume increased with the rejuvenation.

### 3.3. Relationship between geometrical structure and excited regions

**Figure 5.**
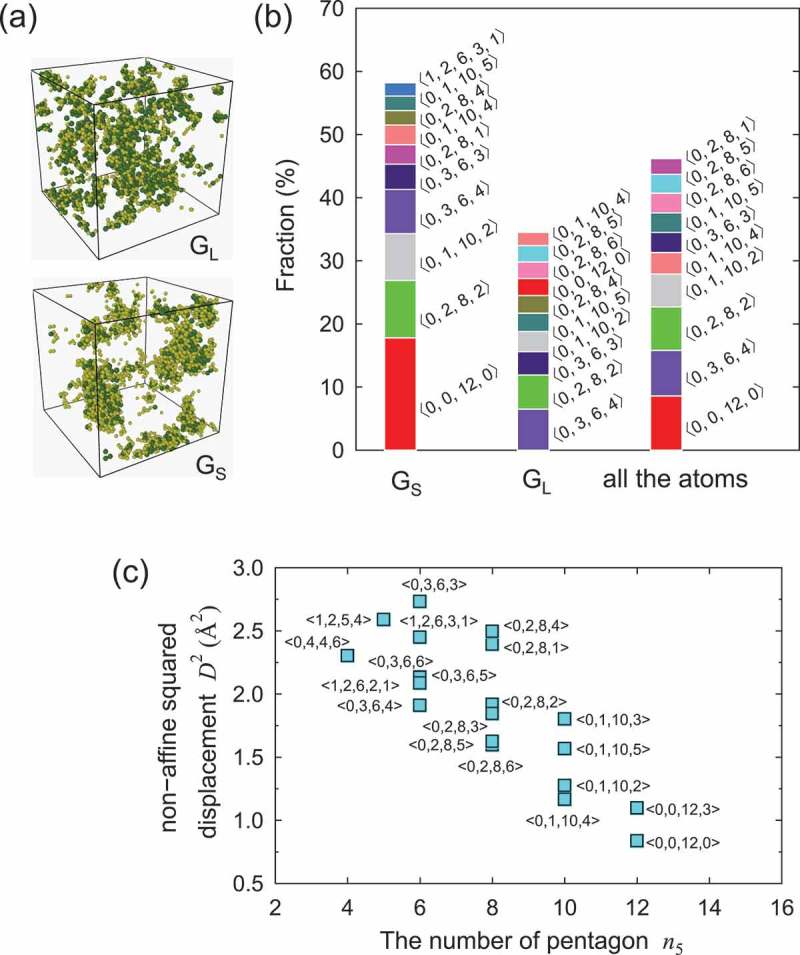
(a) Spatial distribution of atoms with the smallest 5% and largest 5% of $D^{2}$ (i.e., G${\;}_{S}$ and G${\;}_{L}$) in a quenched structure obtained from the snapshot at $t-t_{0}=0.1$ ns. (b) Fraction of 10 major Voronoi polyhedra observed in G${\;}_{S}$, G${\;}_{L}$, and all the atoms. (c) Average $D^{2}$ of 20 major polyhedra for all the atoms. The horizontal axis represents the number of pentagons, $n_{5}$, of each polyhedron.

Figure 5 shows the relationship between the geometrical structure and the less/more-excited regions. We analyzed $D^{2}$ and the geometrical structure of the model quenched from the snapshot at $t-t_{0}=0.1$ ns. Atoms with the smallest and largest 5% of $D^{2}$ were chosen from the quenched model, and labeled group G${\;}_{S}$ and G${\;}_{L}$, respectively. The spatial distributions of the atoms belonging to G${\;}_{S}$ and G${\;}_{L}$ are shown in Figure 5(a). Both the less-excited (i.e., G${\;}_{S}$) and more-excited atoms (i.e., G${\;}_{L}$) formed clusters at the nanometer scale. In other words, the distributions of the less- and more-excited atoms are not homogeneous but heterogeneous.

Figure 5(b) shows the fractions of 10 major Voronoi polyhedra [25] of atoms belonging to G${\;}_{S}$ and G${\;}_{L}$. For comparison, we represent the fractions of the polyhedra of all the atoms. Voronoi polyhedral analysis provides information on the geometric features of the surrounding nearest atoms. The Voronoi polyhedron is denoted by $<n_{3},\;n_{4},\;n_{5},\;n_{6},\;n_{7},\;\cdots >$, where $n_{i}$ represents the number of i-gons. We focused on three major polyhedra, <0,0,12,0>, <0,3,6,4>, and <0,2,8,2>. Icosahedral clusters, denoted by <0,0,12,0>, occupied 8.6% of all the atoms. Because local icosahedral structures exhibit relatively energetic stabilities and high local densities, they have been identified as an important local atomic order in metallic glasses [26,27]. The atoms with the smallest 5% and largest 5% of $D^{2}$ (i.e., G${\;}_{S}$ and G${\;}_{L}$) contain icosahedral clusters of 17.8% and 2.7%, respectively. This result clearly reveals that local regions with high numbers of icosahedral clusters have small $D^{2}$, while local regions with less icosahedral clusters have large $D^{2}$. Meanwhile, the <0,3,6,4> polyhedron, which has been indicated as a distorted fcc structure [28], occupied 7.0%, 6.5%, and 7.2% in G${\;}_{S}$, G${\;}_{L}$, and all the atoms, respectively. The <0,2,8,2> polyhedron, indicated as a distorted icosahedral cluster, occupied 9.1%, 5.4%, and 6.9% of G${\;}_{S}$, G${\;}_{L}$, and all the atoms, respectively. Therefore, among the three major polyhedra, the icosahedral cluster exhibited the largest difference among G${\;}_{S}$, G${\;}_{L}$, and all the atoms. The relationship between the average $D^{2}$ and pentagonal number, $n_{5}$, of each polyhedron is shown in Figure 5(c). A tendency was shown where polyhedra with larger $n_{5}$ had smaller $D^{2}$, and the icosahedral cluster ($n_{5}=12$) had the smallest $D^{2}$, indicating $n_{5}$ (i.e., five-fold symmetry) was correlated with the local structural excitation. On the other hand, we confirmed no clear correlation between $D^{2}$ and the coordination number (defined by the number of faces of the Voronoi polyhedron). In our previous study [19], we revealed an inhomogeneous distribution of icosahedral clusters and their medium-range order in Cu-Zr glass models. The heterogeneity in the geometrical features affects the initiation of plastic deformation; local regions with less icosahedral clusters are relatively well activated under the effects of applied shear strain [14]. Similar to local deformation, the present study revealed that the thermal structural excitation was significantly correlated with the geometrical structure. The more-excited regions with large $D^{2}$, the less icosahedral clusters existed, and vice versa.

### 3.4. Evolution of local atomic order correlating to atomic dynamics

**Figure 6.**
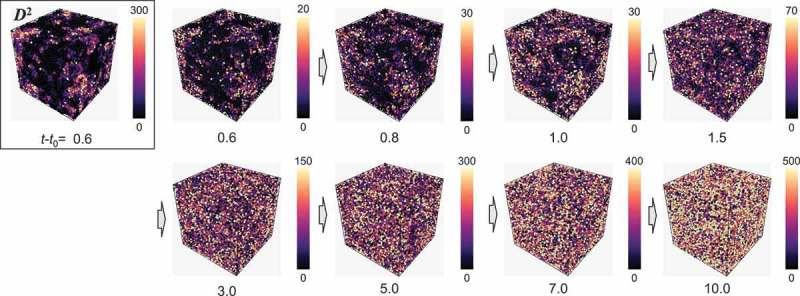
Distribution of atomic non-affine squared displacement at $t-t_{0}=0.6$ ns (upper left-hand corner) and atomic squared displacement for $0.6\leq t-t_{0}\leq 10.0$ ns.

Figure 2 shows the degree of rejuvenation saturation for $2.0\leq t-t_{0}\leq 10.0$ ns. We present the spatial distribution of the atomic non-affine squared displacement $D^{2}$ at $t-t_{0}=0.6$ ns and the atomic squared displacement $\Delta r^{2}$ for $0.6\leq t-t_{0}\leq 10.0$ ns in Figure 6. Again, the atomic squared displacement $\Delta r^{2}$ is defined by the squared displacement of each atom between times $t_{0}$ and t. Therefore, it presents dynamics or motion of individual atoms during the subsequent thermal process. At $t-t_{0}=0.6$ ns, the atomic non-affine squared displacement and the atomic squared displacement exhibited similar distributions. As the structural change in the saturation stage was expected to be large, we used the atomic squared displacement to present the distribution of structural evolution in the saturation stage. In the early stage of the thermal process, the distribution of $\Delta r^{2}$ was heterogeneous as discussed in the previous parts. Subsequently, for $t-t_{0}>3.0$ ns, the atomic squared displacement became larger in the whole model. Therefore, the structural excitation transitioned from partial to whole in the isothermal process. Meanwhile, the atomic squared displacement of each atom exhibited a large difference of up to 10 times or more in the isothermal process, implying that the significant heterogeneity in the atomic dynamics remained through the isothermal process.

**Figure 7.**
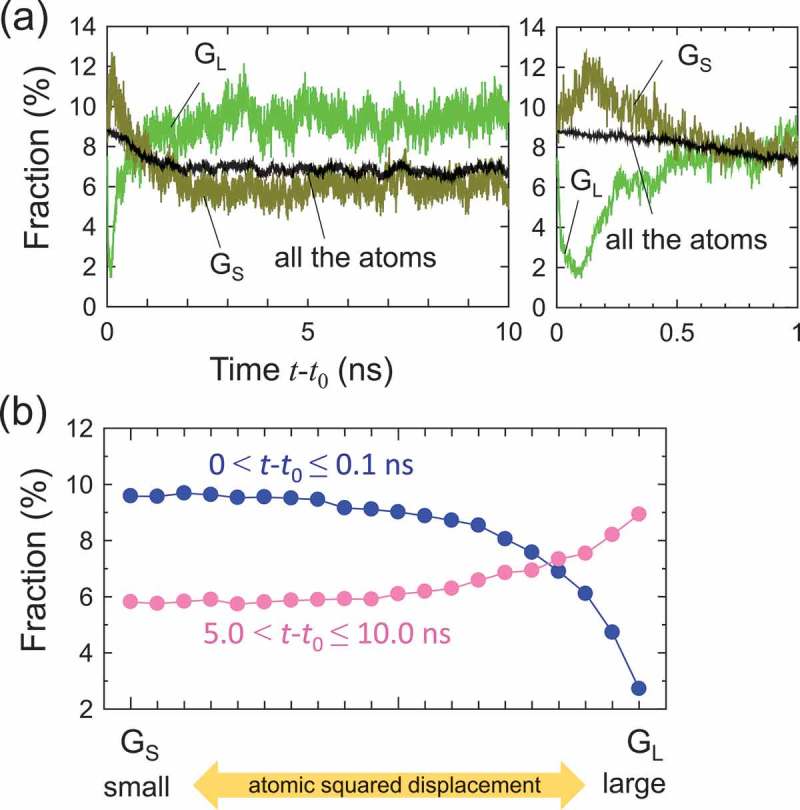
(a) Change in fraction of <0,0,12,0> in atoms with the smallest 5% and largest 5% of $\Delta r^{2}$ and in all the atoms for $0\leq t-t_{0}\leq 10.0$ ns. The enlarged figure of the fraction for $0\leq t-t_{0}\leq 1.0$ ns is shown on the right. (b) Average fraction of <0,0,12,0> with respect to $\Delta r^{2}$ for $0\leq t-t_{0}\leq 0.1$ ns and $5.0\leq t-t_{0}\leq 10.0$ ns. In panel (b), the $\Delta r^{2}$ value increases as from left to right.

Figure 7(a) shows the fractions of <0,0,12,0> in G${\;}_{S}$ and G${\;}_{L}$ for $0<t-t_{0}\leq 10.0$ ns. The fraction of the polyhedron for all the atoms is shown in the figure as well. In Figure 7, atoms with the smallest 5% and largest 5% of $\Delta r^{2}$ were chosen and labeled as G${\;}_{S}$ and G${\;}_{L}$, respectively. The fractions in G${\;}_{S}$, G${\;}_{L}$, and all the atoms demonstrated interesting behaviors during the process. The fraction in all the atoms decreased during the early stage of the rejuvenation process, and became constant in the saturation stage. This trend is reasonable because thermal excitation reduces the local atomic orders, such as the icosahedral structure [5]. Meanwhile, the fractions in G${\;}_{S}$, G${\;}_{L}$ demonstrated characteristic behaviors in Figure 7. As mentioned above, in the very early stage of the thermal process, the less-activated region contained more <0,0,12,0>, and vice versa. The fraction of <0,0,12,0> in G${\;}_{S}$ increased with time and exhibited a peak value (∼12 %) at $t-t_{0}∼0.12$ ns. The peak implies that the correlation between the fraction of the icosahedral structures and the less-excited region, G${\;}_{S}$, becomes significant. After reaching the maximum fraction of icosahedron in G${\;}_{S}$, it decreased as the isothermal process proceeded. This rapid change occurred during $0\leq t-t_{0}\leq 0.1$ ns, which approximately corresponds to the heating period. Meanwhile, the fraction of <0,0,12,0> in G${\;}_{L}$ first decreased but started to increase at $t-t_{0}∼0.1$ ns. A possible reason for this increment is local ordering that occurred in the more-excited regions, resulting in an increase in icosahedral clusters. Subsequently, a crossover between the fractions in G${\;}_{S}$ and G${\;}_{L}$ was observed at $t-t_{0}∼1.0$ ns, and the fraction in G${\;}_{L}$ became larger than that in G${\;}_{S}$ after the crossover. In Figure 7(b), all the atoms in the model are classified into 20 groups depending on the atomic squared displacement. The average fractions of <0,0,12,0> in each group for $0\leq t-t_{0}\leq 0.1$ ns and for $5.0\leq t-t_{0}\leq 10.0$ ns are shown. The fraction of <0,0,12,0> exhibited a correlation with $\Delta r^{2}$ even in the later stage (i.e., $5.0\leq t-t_{0}\leq 10.0$ ns), implying that the correlation between the atomic dynamics and geometrical structure remains. In the later stage, atoms with large atomic squared displacements exhibited a larger fraction of <0,0,12,0>, and vice versa. We emphasize that this trend is opposite to that in the early stage, implying that a significant change in the heterogeneous structure occurred during the isothermal process.

Here, the isothermal process for thermal rejuvenation was conducted at 830 K. We investigated the change in local atomic order during quenching from a high-temperature liquid to low-temperature glass solid (see Figure S1 in Supplementary Materials). The supercooled liquid at 830 K exhibited a lower atomic order than a glass solid but a higher atomic order than a high-temperature liquid. Therefore, after the isothermal process, the alloy had lower atomic order than the relaxed model but higher atomic order than a high-temperature liquid. Figures 2 and 3 indicate that the glass model required approximately 2.0 ns to reach the equilibrium state at 830 K through the rapid heating and isothermal processes. Moreover, because the heating and isothermal processes erase the thermal history and form a new local atomic order, as implied in Figure 7, structural changes during heating and isothermal loading affects the microstructure of the rejuvenated glass.

The crossover in the fraction of the icosahedral structure between G${\;}_{L}$ and G${\;}_{S}$ in Figure 7 can be explained below. In the early stage of the subsequent thermal process, regions of the less-ordered structure were activated earlier than regions of the more-ordered structure, as shown in Figure 5. Subsequently, local ordering occurred in the early activated regions (i.e., less-ordered regions), because the regions exhibited large atomic mobility and large free volume, that promoted relaxation. Meanwhile, local disordering of the later activated regions (i.e., more-ordered regions) occurred, because the amount of local order at 830 K is limited. The increase in the fraction of the icosahedron at regions with large atomic displacements and the decrease at regions with small atomic displacements suggest complex and heterogeneous local ordering and disordering occurred when the system reached equilibrium at 830 K, even though disordering overcame ordering on the average.

### 3.5. Shear deformation analysis

**Figure 8.**
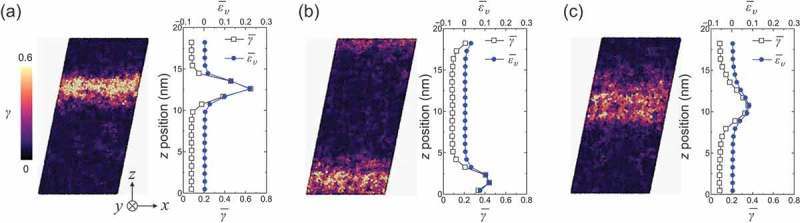
Snapshots of shear simulations at 0.2 applied shear strain. Figure (a) is the relaxed model, M${\;}_{relaxed}$, figure (b) is the rejuvenated model, M${\;}_{5}$, and figure (c) is the rejuvenated model, M${\;}_{6}$. The atomic color represents atomic shear strain γ. The average atomic shear strain $\bar{\gamma }$ and volumetric strain ${\bar{\varepsilon }}_{\upsilon }$ along the z direction are shown on the right. The atomic strains were calculated form snapshots at 10 K without conducting the quenching procedure.

The correlation between structural heterogeneity and deformability, especially in the shear banding behavior, has attracted significant attention in the field of metallic glasses [29–32]. Because rejuvenation changes the microstructure of metallic glasses, rejuvenation affects deformation behaviors [7]. To demonstrate effects of degree of rejuvenation on the deformation behaviors in this model system, we conducted shear simulations. Models with 100,000 atoms were constructed from the relaxed and rejuvenated models (M${\;}_{relaxed}$ and M${\;}_{5}$, respectively). Furthermore, we prepared another rejuvenated model (herein denoted by M${\;}_{6}$) by the subsequent thermal process, in which the isothermal process was conducted at 1000 K for 2 ns. The rejuvenated model M${\;}_{6}$ exhibits a higher degree of rejuvenation than M${\;}_{5}$ owing to the high heating temperature in the subsequent thermal process; a change in the potential energy, i.e., ΔE of 12.7 meV/atom occurred during the subsequent thermal process, higher than that of M${\;}_{5}$ (see Figure 2). For the relaxed model and two rejuvenated models, shear simulations were conducted at 10 K under periodic boundary conditions with a constant strain rate of $10^{8}$ s${\;}^{-1}$ (for more details of the model construction and shear simulation, see Supplementary Materials). Snapshots of the simulations at 0.2 applied shear strain are shown in Figure 8. The atoms are colored based on their atomic shear strain γ. The average atomic shear strains $\bar{\gamma }$ and volumetric strain ${\bar{\varepsilon }}_{\upsilon }$ along the z direction are shown on the right. In all the models, shear strain localized into a band region parallel to the shear direction, although localization occurred at different positions in the z direction owing to the periodic boundary condition. Figure 8 shows that the degree of shear localization was suppressed by rejuvenation: the maximum value of the average atomic shear strain $\bar{\gamma }$ was reduced by rejuvenation, while the thickness (or width in z direction) of the localized region was increased by rejuvenation. The regions with large shear strain exhibit large volumetric strain. Compared with M${\;}_{5}$ and M${\;}_{6}$, M${\;}_{5}$ demonstrated a more localized shear than M${\;}_{6}$, implying that the more rejuvenated model exhibited a more suppressed shear localization. In our previous studies, we have shown that thermal rejuvenation induced more homogeneous deformations [4,5]. The present model system also demonstrated suppressed shear localization by rejuvenation. In addition the present calculation exhibits suppressed local dilatation; the local dilatation is one of factors weakening atomic bondings and softening glass matrix. In the present study, we showed that the microstructure of metallic glasses altered in the thermal rejuvenation process. Therefore, the shear localization behaviors seen in Figure 8 were caused by the change in microstructure by rejuvenation.

## 4. Discussions

**Figure 9.**
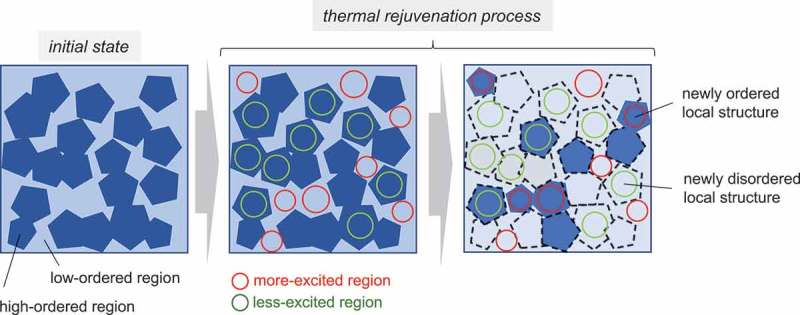
Schematics of the structural heterogeneity and the thermal rejuvenation process predicted in this calculation. The left image shows the microstructures of the initial state, while the center and right images show the microstructures in the rejuvenation process. High-ordered regions contain large numbers of icosahedral clusters (and five-fold symmetry), and the reverse is true. The color of the glass state represents local atomic orders; darker and lighter colors indicate higher and lower atomic orders, respectively. Red and green circles represent more- and less-excited regions, respectively. In the right image, newly ordered local structure at regions with large atomic displacements (or more-excited regions) and newly disordered local structures at regions with small atomic displacements (or less-excited regions) are depicted.

Recently, the heterogeneity in metallic glasses has attracted considerable attention, namely the crystallization behavior (mechanical activation behavior), elastic behavior, thermal strain, and atomic dynamics in supercooled liquids have been studied [8,15,16,18,33]. Experimental studies have indicated that thermal rejuvenation increased β-relaxation regions, suggesting that rejuvenation increased weakly bonding regions [11]. Atomistic approaches have also suggested structural heterogeneity in supercooled liquid and glass [34–36]. The result in this study agrees with the experimental report [11] because rejuvenation increases the free volume (i.e., increases loosely packed regions) and decreases the icosahedral cluster, which is a closely packed local structure. Moreover, our results provided information regarding thermal rejuvenation process, and the correlation among the structural heterogeneity, local atomic orders, and excited regions. In thermal rejuvenation, the structural excitation partially or completely erase the relaxation state of glass, which is determined by the previous thermal treatment, such as the melt-quenching process [5]. As the present study suggests that the structural excitation in the early stage of the isothermal process is heterogeneous, heterogeneity exists in the erase process. Figure 9 shows the schematics of the structural changes in the thermal rejuvenation process predicted in this calculation. In our previous study [14,19], we revealed the inhomogeneous distribution of icosahedral clusters and their medium-range order, implying that metallic glass exhibited a heterogeneous local atomic order (i.e., high and low ordered regions) at the nanometer scale. This study demonstrated that the heterogeneous structural excitation in the early stage was correlated with the local atomic orders; more-excited regions had less icosahedral clusters (i.e., low atomic order) compared with the model average, and vice versa. Several studies have indicated the existence of liquid-like regions (or weakly bonding regions, loosely packed regions) in metallic glass [15,18]. Atoms in these regions are easily activated in the thermal processes owing to the low activation energy barrier or weak atomic bonding, suggesting that these regions correspond to the more-excited regions (i.e., low-ordered regions) in our analysis.

The atomic dynamics in the isothermal process also exhibited a high heterogeneity, and correlated to the local atomic order; the local atomic order increased at regions with large atomic displacements, but decreased at regions with small atomic displacements. This result implies the formation of newly ordered local structures at regions with large atomic displacement and newly disordered local structure at regions with small atomic displacements in the rejuvenation process, although rejuvenation decreased the ordered structure and increased the free volume. These complex structure evolutions in the thermal rejuvenation process indicate a redistribution (or reconstruction) of high- and low-ordered regions (i.e., closely and loosely packed regions, respectively) accompanying significant changes in the heterogeneous microstructure of metallic glasses.

Note that thermal rejuvenation can be realized by increasing the temperature to above $1.1T_{g}$, followed by quenching with faster cooling compared with the previous quenching process. The former process is required for realizing a high-energy state, while the latter is required for suppressing structural relaxation during the subsequent quenching. In this study, we provide atomistic insights into the former process. Furthermore, the structure changes in the final cooling process also affected the microstructure of the rejuvenated glass; this will be investigated further in the future. Metallic glass has been attracted recent attention for applications in nanomolding [37], in which metallic glass is embossed at a temperature above $T_{g}$. As this study provides the atomistic mechanism and features of the local structural changes in metallic glasses by a thermal process above $T_{g}$, the obtained results should also provide fundamental information for practical thermal processes.

## 5. Conclusions

Heterogeneous structural excitation in the rejuvenation of Cu-Zr metallic glass by a thermal process above $T_{g}$ was revealed in this study. During an isothermal process above $T_{g}$, thermally activated atoms were not randomly distributed in the glass matrix, but formed clusters at the nanometer scale, implying that the excitation occurred heterogeneously during the isothermal process. The excited region exhibited large non-affine and affine transformations accompanying the macroscopic potential energy and volume increments. The less-excited and more-excited regions exhibited significant correlations with the icosahedral structure (and five-fold symmetry). The structural excitation transitioned from partial to whole during the thermal rejuvenation process. Although rejuvenation decreased the ordered structure on the average, our calculation results suggested that the rejuvenation process induced newly ordered local structures and newly disordered local structure correlated to local excitations and atomic dynamics, implying the redistribution of the heterogeneous local atomic order in metallic glasses. The changes in the microstructure by rejuvenation affected the degree of shear localization under applied shear deformation.

## Supplementary Materials

The supplementary material for this article can be accessed here.
