# Supplementary material for: Heterogeneous structural changes correlated to local atomic order in thermal rejuvenation process of Cu-Zr metallic glass
Source: Sci Technol Adv Mater. 2019 Jun 19;20(1):632–42. doi: 10.1080/14686996.2019.1624140 (PMC6586098; doi:10.1080/14686996.2019.1624140)
Supplement: Supplemental Material [file TSTA_A_1624140_SM1754.pdf]

Supplementary Materials

Heterogeneous structural changes correlated to local atomic order in thermal rejuvenation process of Cu-Zr metallic glass

Masato Wakeda[a,*], Junji Saida[b]

[a] Research Center for Structural Materials (RCSM), National Institute for Materials Science, Ibaraki 305-0047, Japan

[b] Frontier Research Institute for Interdisciplinary Sciences, Tohoku University, Aramaki aza Aoba 6-3, Aoba-ku, Sendai 980-8578, Japan

*WAKEDA.Masato@nims.go.jp

1. Changes in potential energy and local order in a quenching process

We evaluated changes in the potential energy and local atomic order during a melt-quenching process. Using atomic models with 10,000 atoms, we conducted melt-quenching simulations from 2000 K to 0 K with a constant cooling rate of 10^{11} K/s. To remove the effects of atomic thermal fluctuations on the calculation of potential energy and local atomic order, we used atomic configurations, which were instantaneously quenched from snapshots of the melt-quenching process. Here the instantaneously quenching was realized using the conjugate gradient method, in which the model size was controlled to keep a zero-pressure condition. We conducted three independent simulations with different initial atomic configurations and velocities and calculated average values of three models. The averaged changes in the potential energy and local atomic order are shown in Figure S1. The potential energy of the liquid gradually decreased during the quenching process. Then below 1100 K the potential energy showed a drastic decrement because the liquid alloy became a supercooled liquid, which had higher local atomic order than high-temperature liquid as seen in Figure S1 (b). Below T_g, changes in the potential energy and the local atomic order became small, because the system froze into glass solid. Figure S1 indicates the thermal history between 1100 K and T_g has a crucial effect on the construction of microstructure in the present alloy. For thermal rejuvenation, we employed the thermal process at 830 K, which was higher than T_g but lower than 1100 K. Therefore, the supercooled liquid at 830 K had higher local atomic order than high-temperature liquid as seen in Figure S1 (b).

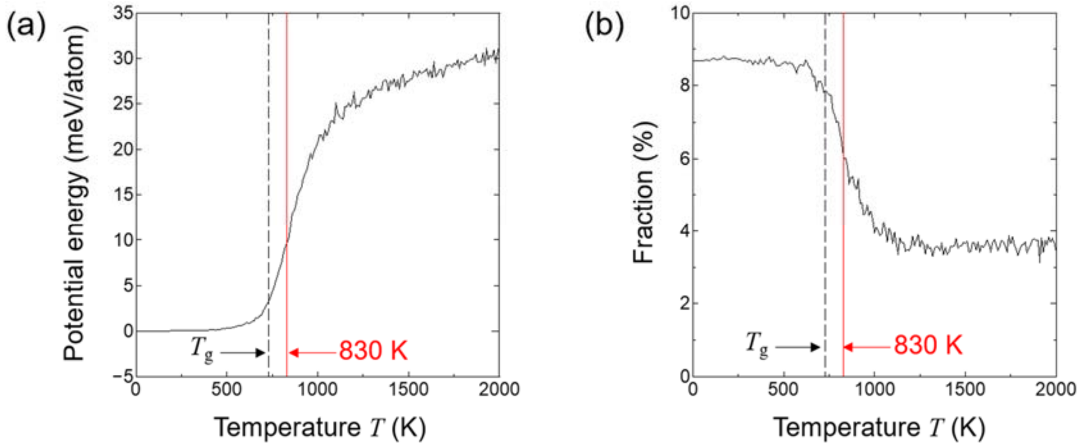

Figure S1 Changes in (a) potential energy and (b) fraction of icosahedral structure during a quenching process from 2000 K to 0 K (a cooling rate is 10^{11} K/s). T_g determined by a change in slope of the volume-temperature curve in the quenching process is depicted as a vertical broken line (black). The temperature of the isothermal heat treatment (i.e., 830 K) is also depicted as a vertical line (red). In figure (a) the potential energy at 0 K was used as a reference energy state (i.e., 0 meV/atom).

2. Shear simulations of the relaxed and rejuvenated models

We conducted shear simulations of the relaxed and rejuvenation models. For the shear simulations, we prepared models with 100,000 atoms as follows. The relaxed and rejuvenated model ($M_{relaxed}$ and M_5, respectively) with 50,000 atoms were used as unit models. Using the unit models, we constructed large relaxed and rejuvenated models with 100,000 atoms ($1 \times 1 \times 2$ array of the unit model). We also prepared another rejuvenated model (herein denoted by M_6) by the subsequent thermal process, in which the isothermal process was conducted at 1000 K and for 2.0 ns (i.e., $l_6 = 2.0$ ns). The rejuvenated model M_6 exhibits a higher degree of rejuvenation than M_5 owing to the higher temperature in the subsequent thermal process. Before shear loading, the relaxed model and two rejuvenated models with 100,000 atoms were heated to 300 K, relaxed at 300 K for 0.1 ns, and cooled to 10 K (both the heating and cooling rates were 10^{13} K/s). For the relaxed model and rejuvenated models, shear simulations were conducted at 10 K under periodic boundary conditions with a constant strain rate of 10^8 s^{-1}. During the shear simulations, we fixed the height of the models (i.e., the *z* dimension).
